# A New Insight into the Potential Role of Tryptophan-Derived AhR Ligands in Skin Physiological and Pathological Processes

**DOI:** 10.3390/ijms22031104

**Published:** 2021-01-22

**Authors:** Monika Szelest, Katarzyna Walczak, Tomasz Plech

**Affiliations:** Department of Pharmacology, Medical University of Lublin, Chodźki 4a, 20-093 Lublin, Poland; m.wlodarczyk214@gmail.com (M.S.); tomasz.plech@umlub.pl (T.P.)

**Keywords:** aryl hydrocarbon receptor, tryptophan, kynurenine, FICZ, skin, kynurenic acid, atopic dermatitis, psoriasis, melanoma

## Abstract

The aryl hydrocarbon receptor (AhR) plays a crucial role in environmental responses and xenobiotic metabolism, as it controls the transcription profiles of several genes in a ligand-specific and cell-type-specific manner. Various barrier tissues, including skin, display the expression of AhR. Recent studies revealed multiple roles of AhR in skin physiology and disease, including melanogenesis, inflammation and cancer. Tryptophan metabolites are distinguished among the groups of natural and synthetic AhR ligands, and these include kynurenine, kynurenic acid and 6-formylindolo[3,2-b]carbazole (FICZ). Tryptophan derivatives can affect and regulate a variety of signaling pathways. Thus, the interest in how these substances influence physiological and pathological processes in the skin is expanding rapidly. The widespread presence of these substances and potential continuous exposure of the skin to their biological effects indicate the important role of AhR and its ligands in the prevention, pathogenesis and progression of skin diseases. In this review, we summarize the current knowledge of AhR in skin physiology. Moreover, we discuss the role of AhR in skin pathological processes, including inflammatory skin diseases, pigmentation disorders and cancer. Finally, the impact of FICZ, kynurenic acid, and kynurenine on physiological and pathological processes in the skin is considered. However, the mechanisms of how AhR regulates skin function require further investigation.

## 1. Introduction

The aryl hydrocarbon receptor (AhR) is expressed in various tissues characterized by a rapid growth rate, including skin [1]. Gene expression analysis revealed that AhR activation enhances or suppresses the expression of several genes, thus influencing the gene expression profile [2]. Previous studies revealed the crucial role of AhR in several physiological and pathological processes in the skin. Among the groups of natural and synthetic AhR ligands is the group of tryptophan derivatives [1,3]. Some of them, including kynurenine, kynurenic acid, and 6-formylindolo[3,2-b]carbazole (FICZ), have been previously recognized as ligands of this receptor. However, recently discovered biological properties of these substances, their widespread presence, and potential continuous exposure may suggest the important role of tryptophan-derived AhR ligands in many physiological and pathological processes in the skin [4]. Unfortunately, the role of AhR itself and the biological effect of the tryptophan-derived ligands in the prevention, pathogenesis, and progression of skin diseases are not fully understood to date.

## 2. Aryl Hydrocarbon Receptor (AhR)

AhR is a transcription factor from the evolutionarily old family of a basic helix-loop-helix/Per-ARNT-Sim (bHLH-PAS) transcription regulators, acting in a DNA sequence-specific manner. The bHLH motif contains two domains that are responsible for DNA sequence binding and protein dimerization [1]. Several low-molecular-weight chemical compounds activate the cytosolic AhR after entering cells via diffusion [3,5]. Air pollution compounds [3], endogenous amino acid derivatives [6,7,8], some food components (e.g., indoles, polyphenols, glucosinolates) [9,10,11], and some yeast and bacterial metabolites [12] are considered AhR ligands. AhR has only one binding pocket, whose amino acid composition determines ligand binding strength [1]; however, AhR may also be activated by a number of stress factors and some substances that might not fit into the binding pocket (e.g., hypoxia and oxidized low-density lipoproteins) [13].

The type of AhR ligand determines the level of activation and the spectrum of genes transcribed [14,15,16]. An increased AHR expression is observed in the placenta, liver, lungs, intestines, and skin, which are barrier tissues or play an important role in metabolism. The lowest *AHR* expression is reported in the brain, kidneys, and skeletal muscles [2]. Recent studies revealed additional functions of AhR in the body, including control of liver and vascular development, intestinal immunity, hematopoiesis, and perinatal growth [17,18,19,20,21,22]. Moreover, AhR signaling may be associated with stem cell proliferation and carcinogenesis [2].

**Figure 1:**
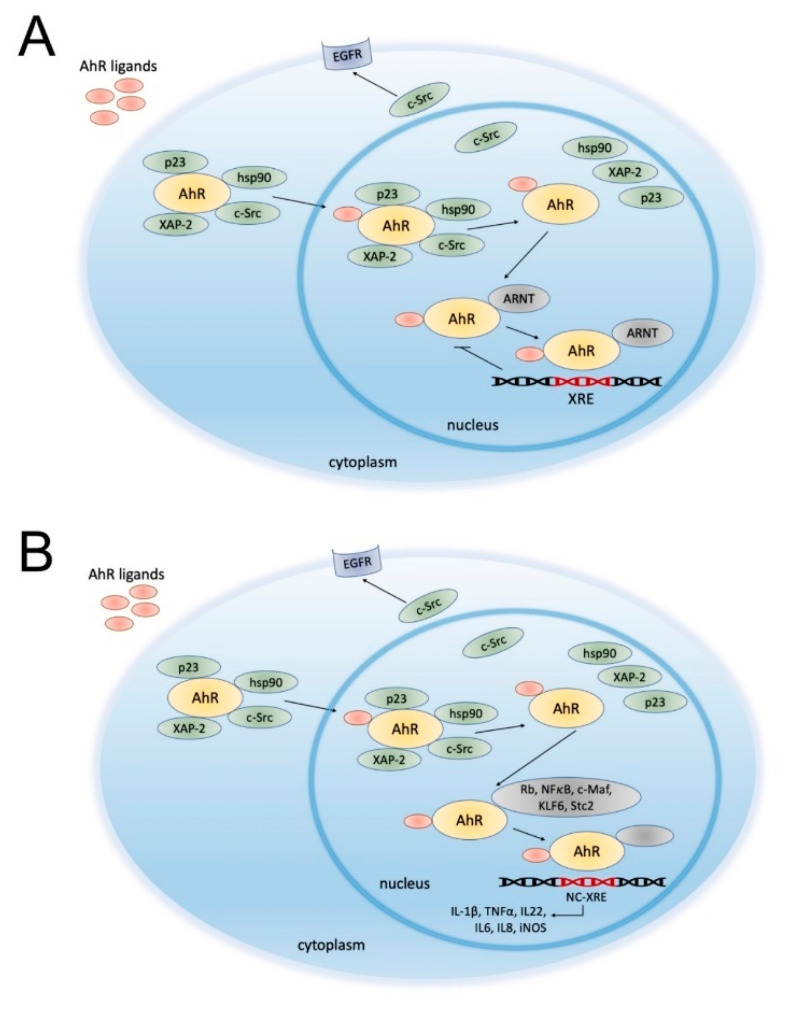
Schematic overview of aryl hydrocarbon receptor (AhR) signaling pathways: canonical (**A**) and noncanonical (**B**). In physiological conditions, AhR is localized in the cytosol and complexed with specific proteins, such as the hepatitis B virus X-associated protein 2 (XAP-2), heat shock protein 90 (HSP90), c-Src and p23. Upon ligand binding, AhR changes its conformation and is translocated to the nucleus, where it dimerizes with AhR nuclear transporter (ARNT) (**A**) or other partners, such as transcription factors (e.g., Kruppel-like factor 6 (KLF6)). (**B**). Dissociated c-Src interacts with the epidermal growth factor receptor (EGFR). To date, several different types of crosstalk between AhR and other proteins have been described. For instance, AhR interaction with the hyperphosphorylated form of the retinoblastoma protein (Rb) results in growth arrest at the G1/S phase of the cell cycle [24]. AhR signaling may also promote nuclear factor kappa-light-chain-enhancer of activated B cell (NF-κB) activation via RelA and RelB interaction [25,26,27]. Moreover, AhR signaling is associated with the activity and function of the estrogen receptor [28]. The AhR/ARNT complex binds to the xenobiotic-responsive element (XRE) and induces the transcription of AhR-responsive genes (e.g., *CYP1A1*). On the other hand, AhR ligation promotes the transcription of its inhibitor—the AhR repressor (AhRR). AhRR forms a heterodimer with ARNT and competes with AhR/ARNT to bind to the XRE sequence, inhibiting AhR-induced transcription. However, previous studies suggest that AhR repression may not occur solely by inhibition of the DNA binding site and AhR/ARNT complex formation [29,30]. Moreover, Wilson et al. indicated that AhR–KLF6 complex formation may be involved in cell cycle regulation [31]. AhR and KLF6 proteins form a heterodimer that recognizes novel nonconsensus XRE (NC-XRE), highlighting a distinction from the XRE-dependent AhR signaling mechanism. This noncanonical signaling pathway may influence cell cycle regulation, as it controls the expression of the cyclin-dependent kinase inhibitor p21Waf1/Cip1 [31]. Jackson et al. revealed that 2,3,7,8-tetrachlorodibenzo-p-dioxin (TCDD)-mediated p21Waf1/Cip1 activation is associated with disrupted liver regeneration [32]. Therefore, while the KLF6-mediated noncanonical AhR signaling pathway might suppress tumor growth by regulating p21Waf1/Cip1 expression, carcinogenic AhR agonists might activate the canonical AhR signaling pathway and promote tumorigenesis. As outlined above, AhR might influence cell survival by various mechanisms. However, AhR might also interact with different genes that have a similar binding pattern, such as *STC2* gene, encoding a glycoprotein responsible for the regulation of endoplasmic reticulum stress [33]. Vogel et al. revealed that AhR forms a complex with NF-κB subunit RelB. The NF-κB-RelB-binding site is targeted by AhR and promotes the expression of chemokine genes, such as *BAFF*, *BLC*, and *IRF3* [34]. Furthermore, Ge et al. identified Rb as an AhR dimerization partner, suggesting its role in cell cycle arrest [35]. Recently, Huang et al. described a novel NC-XRE in the promoter of the gene encoding plasminogen activator inhibitor-1 (*PAI-1*) that might be targeted by a distinct protein complex [36]. However, further investigations are needed to determine the contribution of canonical and noncanonical AhR signaling pathways to cell homeostasis. The scheme is based on previously reported data [2,3,14].

After ligand binding, AhR dissociates from its chaperones (e.g., proto-oncogene tyrosine-protein kinase c-Src, heat shock protein 90 (HSP90), p23, and the hepatitis B virus X-associated protein 2 (XAP2)) and undergoes conformational changes, resulting in the exposition of the nuclear translocation signal and induction of AhR transport into the nucleus (Figure 1A) [23].

Chaperone c-Src disconnected from AhR initiates internalization and nuclear translocation of the epidermal growth factor receptor (EGFR) and activation of mitogen-activated protein kinases (MAPK) signaling cascades, which are involved in cell proliferation, migration, and angiogenesis [37]. In the nucleus, AhR dimerizes with the AhR nuclear transporter (ARNT), another member of the bHLH-PAS family [38]. Genes possessing AhR binding sites (xenobiotic response elements, XRE) in their promoters are transcribed when bound to an AhR/ARNT dimer. After the interaction with XRE, AhR is transported back into the cytosol and degraded. The crosstalk between AhR and other signaling pathways may modify the effects of AhR and its ligands’ interaction (Figure 1B) [1].

The wide spectrum of genes interacting with AhR within XRE indicates that AhR signaling is specific to the type of cell, tissue, or prevailing conditions (Table 1) [15,16].

The transcription of genes encoding xenobiotic-metabolizing enzymes (*CYP1A1, CYP1A2*, and *CYP1B1*), genes responsible for cell differentiation and regulation of the cell cycle, and genes coordinating the immune response is dependent, even partially, on AhR activity [65,66]. Moreover, induction of *CYP1A1* expression allows for the degradation of some AhR ligands, including FICZ. During this process, a large amount of reactive oxygen species (ROS) is produced. ROS activity affects cell metabolism, leading to DNA damage and expression of various cytokines [65,67].

Interestingly, microarray studies indicated a ligand-specific differences in AhR-induced gene expression profile [68]. To date, many cellular metabolites and xenobiotic compounds were defined as AhR agonists [2,67].

The mechanism of downregulation of AhR signaling is still unclear. It is based on the activity of the negative AhR regulator, the AhR repressor (AhRR). Upon AhR activation, *AHRR* expression is induced. The AhRR forms a heterodimer with ARNT and competes with the AhR/ARNT complex for the XRE binding site. This feedback loop consequently inhibits AhR transcriptional activity [69]. On the other hand, Evans et al. reported that AhRR-mediated AhR inhibition is not the cause of ARNT sequestration [29]. A distinct mechanism of AhRR action was proposed, indicating that AhR inhibition occurs through protein–protein interaction [29]. Therefore, AhR repression does not occur solely by inhibition of the DNA binding site and AhR/ARNT complex formation.

## 3. The Role of AhR in Skin Physiology

Skin, the largest organ of the human body, is a protective barrier against harmful environmental factors. The maintenance of body fluid balance and a constant temperature depends on the proper condition and function of the skin. A battery of receptors and nerve endings present in the skin enable a reaction to various stimuli and communication with the surrounding environment [70].

The skin has a layered structure, consisting of (from the outside): epidermis, formed mainly by keratinocytes; dermis, created mainly by fibroblasts; and subcutaneous tissue. Among skin cells, there are also Langerhans cells (LCs), melanocytes, sebocytes, and immune cells (mast cells, CD8+ T cells, and dendritic cells (DCs)) [70]. AhR is observed in all skin cells, but particular cell types differ in its expression level [2].

The skin is exposed to biological, physical, mechanical, and chemical factors. Interestingly, AhR signaling appears to play an important role in maintaining skin homeostasis as it participates in many processes such as metabolism of environmental toxins, maintaining redox balance in the cell, response to ultraviolet (UV) radiation, melanogenesis, regulation of immunological processes, and functioning of the epidermal barrier [2].

AhR/ARNT signaling initiates the activation of the OVO-like 1 (OVOL1) transcription factor, which subsequently enhances the expression of filaggrin (FLG) and loricrin (LOR), proteins specific to fully differentiated keratinocytes and corneocytes [71]. Thus, the activation of this pathway contributes to accelerating the final differentiation of the epidermis and formation of epidermal barrier.

The role of the skin in immune processes is based on protecting the host from pathogens while suppressing excessive inflammation. High levels of AhR in the skin cells may be associated with an AhR-mediated immune response. AhR signaling is essential for the maturation of LCs and its capacity to present antigens, as demonstrated in studies on *AHR*-null mice [72]. Interestingly, inflammatory skin lesions were observed in mice with permanently active AhR in keratinocytes [73]. High *AHR* expression was previously reported in Th17 cells. Moreover, IL-22 secretion by these lymphocytes depends on AhR activation [74]. In summary, AhR deficiency or alterations within AhR activity may disrupt the immune response or impair the development and function of the epidermal barrier [75].

## 4. AhR and Skin Pathological Processes

In addition to the prominent and well-documented role of AhR in skin homeostasis, this receptor is also involved in many pathological processes within the skin through alterations in AhR-controlled signaling pathways. Moreover, it may be associated with exposure to toxic AhR ligands present in air pollution. Disorders whose pathomechanism is associated with AhR function in the skin include, among others, chloracne, hyperpigmentation, and vitiligo, as well as inflammatory diseases such as psoriasis or atopic dermatitis [66].

Skin diseases may be related to air pollution. The most common air pollutants with high affinity for AhR include the following: 2,3,7,8-tetrachlorodibenzo-p-dioxin (TCDD), benzo[a]pyrene (BaP), polychlorinated dibenzofurans (PCDFs), polychlorinated dibenzo-p-dioxins (PCDDs), and polychlorinated biphenyls (PCBs) [2,66]. Although exposure to high doses of toxic AhR ligands is relatively rare and accidental, even low doses of these compounds have previously caused skin irritation or worsened the symptoms of diseases [76]. Chloracne and hyperpigmentation are the most frequently mentioned among skin diseases in people exposed to high doses of air pollution components [77,78,79,80]. AhR may also be activated due to chronic exposure to PM2.5, which is made up of dioxin derivatives [76].

Activation of the AhR signaling pathway in epidermal keratinocytes is sufficient to initiate inflammatory skin lesions [73]. Tauchi et al. suggest that the activation of the AhR signaling pathway and the expression of AhR target genes are the main mechanisms of inflammatory skin disorders induced by PAH [73]. Thus, blocking of AhR signals that induce transcription of selected genes may be a potential therapeutic target in the treatment of some skin diseases.

### A Double Agent: The Role of AhR in Oxidative Stress

Oxygen molecules do not always undergo full four-electron reduction, which leads to the formation of unstable ROS. In physiological conditions, ROS are formed during biochemical reactions and are characterized by high reactivity. Moreover, ROS are produced as a result of exposure to environmental stress, such as UV radiation or ionizing radiation. Xenobiotics and air pollutants may also increase ROS formation. The balance between the rate of ROS formation and the activity of antioxidants produced by the cells determines the biological response to ROS. The consequences of increased cell exposure to ROS include the following: a decrease in adenosine triphosphate (ATP) levels, lipid peroxidation, cell membrane depolarization, morphological changes in cell surfaces, and DNA damage. However, the biological activity of ROS is not limited to adverse effects, as at a physiological concentration, they play an important role in cell homeostasis by regulation of proliferation, apoptosis, and migration [81].

The interaction of TCDD with AhR enhances the expression of cytochrome P450 family members such as CYP1A1, CYP1A2, and CYP1B1. Due to the stable structure of TCDD, these enzymes are not able to metabolize TCDD dioxin effectively. Furthermore, excessive CYP1A1 activity, resulting from the constant interaction between TCDD and AhR, induces the generation of ROS. Increasing oxidative stress may cause oxidation of fatty acids in skin cell membranes, structural proteins (mainly collagen), and enzymatic proteins [14]. CYP1A1-induced excessive production of ROS may indirectly affect cell metabolism due to direct activation of multiple signaling pathways. Moreover, an interaction of ROS with various molecules such as NF-κB, c-Jun oncoprotein, or Rb may affect the cell cycle [82,83].

Activation of the AhR/CYP1A1 signaling pathway also contributes to increased production of inflammatory mediators, including interleukin 1 (IL-1), IL-6, and IL-8. Furthermore, BaP exposure is associated with an increased expression of *CYP1A1*, and IL-8, ROS production. This phenomenon may underlie the inflammatory skin diseases in tobacco smokers, as BaP is a component of tobacco smoke [84,85].

On the other hand, AhR activity induces the expression of nuclear factor erythroid 2-related factor 2 (Nrf2), a transcription factor with antioxidant properties. Upon AhR-mediated activation, Nrf2 increases the expression of antioxidant enzymes such as glutathione S-transferases and NAD(P)H quinone dehydrogenase 1 (NQO1) [56]. Some AhR ligands are more active in promoting the antioxidant response (Table 2).

Modulation of the activity of various proteins, including downstream AhR targets, such as CYP1A1 or Nrf2 via the AhR/ARNT pathway, determines the redox balance of the cells [56].

In contrast to TCDD, coal tar induces Nrf2 nuclear translocation and follows the induction of *NQO1* expression, thereby triggering an antioxidant signal pathway that neutralizes the negative effect of ROS in keratinocytes [92]. Activation of this pathway may be a clue suggesting the lack of toxicity and carcinogenicity of coal tar used in the treatment of psoriasis [93]. On the other hand, chronic exposure to TCDD results in growing immunotoxicity, thereby increasing the risk of cancer [65]. Although the mechanism of coal tar activity is not fully understood, a comprehensive study in a large group of patients with psoriasis and eczema has not indicated a relationship between the use of coal tar and an increased risk of skin cancers [93]. In summary, both ROS production and antioxidative response resulting from AhR activation depend on the AhR ligand type.

## 5. Role of AhR in Inflammatory Skin Diseases

### 5.1. Atopic Dermatitis

Atopic dermatitis (AD) is a heterogeneous skin disease accompanied by eczema, Th2-deviated inflammation, and chronic itching. Due to the reduced expression of FLG and other proteins involved in the differentiation and maturation of skin cells, the skin barrier integrity in AD is impaired [94]. Moreover, skin barrier dysfunction causes an increased colonization of microorganisms, such as *Staphylococcus aureus*, which further promotes skin inflammation [95].

Previous studies suggested that Th2-mediated immune response is associated with reduced production of the tryptophan-derived AhR ligand indole-3-aldehyde (IAId) by the skin microbiome. Yu et al. reported that IAId-induced AhR activation attenuated AD-like dermatitis [96]. Decreased inflammation was associated with the inhibition of thymic stromal lymphopoietin (TSLP) production in keratinocytes. TSLP is an inflammatory cytokine overexpressed in keratinocytes of AD patients. Upon IAId stimulation, AhR may interact with the *TSLP* promoter region and promote immune homeostasis in the skin of healthy subjects. TSLP expression is also observed in MC903-induced AD-like dermatitis mouse model, as it plays a crucial role in Th2-mediated inflammation. Although the inhibitory effect of IAId on TSLP expression reduces the inflammatory response in MC903-induced AD-like dermatitis in mice, this effect has not been observed in different models of AD-like skin inflammation, such as imiquimod (IMQ)-induced psoriatic dermatitis and oxazolone (OXA)-induced contact hypersensitivity. Due to aberrant skin microbiota, a reduced level of IAId may indicate alterations in TSLP expression, leading to skin inflammation in patients diagnosed with AD. Therefore, a deficiency of physiological AhR ligands in the Th2-deviated environment may underlie the skin lesions in AD [96].

**Figure 2:**
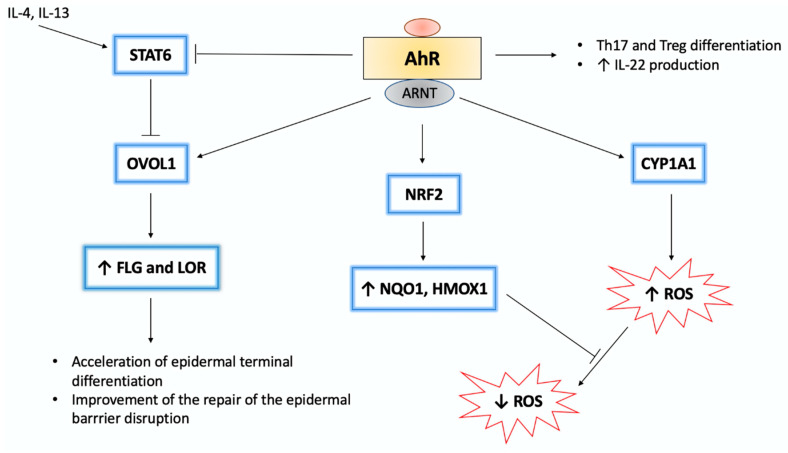
Molecular interactions within the AhR signaling pathway [99]. In the nucleus, AhR/ARNT complex binds to the XRE sequence, thus initiating the transcription of AhR-responsive genes, such as *CYP1A1*. CYP1A1 activity is associated with ROS production. Furthermore, AhR ligands are degraded by CYP1A1. Regarding chemically stable ligands, such as dioxins, sustained CYP1A1 activation leads to enhanced ROS generation. On the other hand, several AhR ligands activate nuclear factor-erythroid 2-related factor-2 (Nrf2), a transcription factor, which induces expression of antioxidative enzymes (e.g., heme oxygenase 1 (HMOX1) and NQO1). Moreover, AhR signaling is associated with the differentiation of immune cells, such as Th17 and Treg. Regarding inflammatory skin diseases, such as psoriasis and atopic dermatitis, AhR-mediated IL-22 production plays a crucial role in alleviating skin lesions. AhR/ARNT interaction upregulates filaggrin (FLG) and loricrin (LOR) expression via activation of the OVO-like 1 (OVOL1) transcription factor. Both FLG and LOR play a key role in epidermal differentiation. However, IL-4/IL-13-mediated signal transducer and activator of transcription 6 (STAT6) activation inhibits the OVOL1/FLG/LOR pathway. AhR stimulation may inhibit STAT6 and upregulate FLG and LOR expression. The pathogenic implication of AhR signaling in inflammatory skin diseases is not fully understood as the activation of the AhR/OVOL1/FLG/LOR pathway may become harmful. As the use of rapid metabolizing AhR ligands, such as FICZ, may alleviate skin inflammation, sustainable activation of this pathway by dioxins exacerbates epidermal barrier dysfunction. The scheme is based on previously reported data [9,10,84,99]. ↑-activation, upregulation, ↓-downregulation, T-arrow-inhibition.

Expression of FLG in keratinocytes is dependent on AhR activity as AhR ligation leads to OVOL1 nuclear translocation and subsequent FLG transcription [62]. The AhR/ARNT/FLG signaling pathway may be activated by both rapidly metabolized AhR ligands, such as IAId or FICZ, and by dioxins (Figure 2) [97,98].

Therefore, dioxin-mediated or persistent AhR activation may promote skin barrier dysfunction and exacerbate the course of AD [97]. However, topically applied FICZ reduced inflammation in skin lesions in a murine dermatitis model by AhR activation [98]. Moreover, a decrease in *Il 22* expression and an increase in *FLG* transcription were observed [98].

However, the role of AhR in AD pathogenesis is not fully understood. Kim et al. showed an increase in *ARNT* and *CYP1A1* messenger RNA (mRNA) expression in AD skin [100]. On the other hand, Hong et al. revealed an increased protein level of AhR and ARNT but not CYP1A1 in skin lesions of AD patients [97]. Hu et al. demonstrated higher expression of *AHR* in serum and increased protein level of AhR in skin lesions of AD patients compared to healthy controls. Moreover, mRNA levels of *AHR, AHRR*, and *CYP1A1* in peripheral blood mononuclear cells (PBMCs) of AD patients were higher in comparison to healthy controls. Thus, *AHR* expression level in PBMCs may be associated with eczema area and severity index score in AD patients [101].

The antioxidative transcription factor Nrf2 may be activated by some AhR ligands, and recent studies indicated a therapeutic effect of this group of AhR agonists. For instance, coal tar attenuates inflammatory response in AD and psoriasis patients by *NRF2* activation upon AhR interaction [92,102]. However, excessive activation of AhR leads to abnormally accelerated keratinization of cells and the formation of pruritic artemin [103,104].

One of genes encoding nerve elongation factors that may be related to epidermal hyperinnervation is *ARNT. ARNT*, encoding artemin, acts as pruritus-related AhR target gene. Edamitsu et al. suggest that besides *ARNT* overexpression, constitutive AhR activation may exacerbate alterations in the epidermis in patients with AD [103]. Moreover, artemin expression and alloknesis may be enhanced by air pollutants via AhR activation [104]. Artemin expression is higher in patients with AD compared to healthy controls [104]. Topical application of 7,12-dimethylbenz[a]anthracene (DMBA), an exogenous AhR agonist, induced an AD-like phenotype, but this effect was not achieved when using endogenous AhR ligand FICZ. As FICZ is rapidly metabolized by CYP1A1, it cannot efficiently activate AD-related target genes. Therefore, prolonged AhR activation is crucial for pruritic AD symptoms induction [104].

A few reports indicate that some AhR agonists, such as FICZ, 2-(1H-Indol-3-ylcarbonyl)-4-thiazolecarboxylic acid methyl ester (ITE), and soybean tar Glyteer, may activate both canonical and noncanonical AhR signaling pathways. For instance, in human keratinocytes, FICZ promotes wound healing via extracellular signal-regulated kinase (ERK) signaling in an AhR-independent manner [105]. AhR endogenous ligand ITE also reduces transforming growth factor-beta (TGF-β) signaling without AhR activation. However, the recruitment of Th2 cells in AD skin lesions is regulated by chemokine (CC motif) ligand 17 (CCL17) and CCL22 expression. Both chemokines are produced via signal transducer and activator of transcription 6 (STAT6) activation in DCs. Takemura et al. demonstrated that soybean tar Glytter inhibits STAT6 expression; thus, CCL17 and CCL22 production in DCs is reduced [106]. Moreover, STAT6 expression is blocked by coal tar via AhR-mediated activation of the Nrf2 signaling pathway [92]. Interestingly, coal tar induces a shift in skin microbiome composition due to the microbiome-modulating properties of some AhR agonists. As the skin microbiome plays an important role in the development of inflammatory skin diseases, this biological mechanism of coal tar may have an essential therapeutic value [107].

Clinical studies confirm the efficacy of the AhR agonist tapinarof in the treatment of AD [108]. The action of tapinarof is based on the activation of the Nrf2-antioxidative pathway. Improvement in skin condition after tapinarof application is also associated with reduced IL-17A production and increased *FLG* expression [108].

### 5.2. Psoriasis

Psoriasis is a chronic inflammatory skin disease characterized by the thickened epidermis and skin infiltration of polymorphonuclear cells. The tumor necrosis factor-alpha (TNF-α)/IL-23/IL-17A axis plays a key role in induction and progression of psoriasis; thus, biological drugs against TNFα/IL-23/IL-17A have good therapeutic efficacy [109].

The interaction between AhR and endogenous ligands changes the inflammatory profile of skin lesions in psoriasis [110]. AhR-mediated Th17 activity controls the expression of IL-22 [111,112]. Monitoring of IL-22 plasma concentration allows the assessment of the severity of the disease [113]. Furthermore, the activity of IL-22 in keratinocytes is associated with increased expression of the transcription factor STAT3, which contributes to increased proliferation of epidermal cells [114]. IL-22 also affects the final stage of epidermal cell differentiation, leading to psoriasis-like skin lesions [115,116].

Interestingly, AhR activity is required for IL-22 production specifically by Th17 cells. AhR induction is not necessary for other types of IL-22-producing cells, including γδ T cells, CD4(−)CD8(−)TCRβ(+) T cells, and innate lymphoid cells. It is still unclear why Th17 specifically requires AhR stimulation to produce IL-22. Nevertheless, the reason for it may indicate the diversity of interactions of AhR downstream effectors with other transcription factors. For instance, TGF-β, which induces c-Maf activity, is involved in the differentiation of Th17 cells. C-Maf inhibits IL-22 expression by binding to its promoter. Hence, AhR activity appears to be necessary to overcome the suppressive activity of TGF-β [115].

On the other hand, the interaction of AhR with endogenous ligand FICZ reduces the inflammatory response in the IMQ-induced model of skin lesions [117]. Moreover, *AHR*-null mice presented significant exacerbation of the disease when compared to the *AHR*-sufficient control. In addition, an increase in mRNA expression of several proinflammatory cytokines involved in psoriasis, such as *Il 17a*, *Il 17c*, *Il 23*, *Il 22*, and *Il 1b*, was observed in the skin lesions of *AHR*-deficient mice [117].

Nevertheless, the role of AhR in psoriasis is controversial [99]. Kim et al. reported an increase in AhR and ARNT protein level in skin lesions in psoriasis, whereas CYP1A1 level was decreased when compared to healthy skin [100]. However, the fact that AhR may induce the expression of other genes not involved in the metabolism of xenobiotics cannot be ignored. It should be underlined that AhR controls activation of several signaling pathways, including phosphoinositide 3-kinase/protein kinase B (PI3K/Akt) and ERK signaling pathways, and the expression of various genes contributing to proliferation, adhesion, migration, or immune response [118,119]. On the other hand, serum levels of AhR and CYP1A1 in psoriasis patients were significantly higher when compared to the control group in the study conducted by Beranek et al. [120].

One of the genes that is found to be consistently upregulated in psoriatic skin lesions is *KYNU*, encoding an enzyme of the tryptophan metabolism. Kynureninase (KYNU) degrades kynurenine, an endogenous AhR ligand [121]. Gudjonsson et al. revealed other genes (e.g., *IDO1*, *CYP2E1*, *CYP4B1*, *SMOX*, and *ALDH3A2*) of the tryptophan catabolism pathway to be differentially regulated in psoriasis [122]. Deregulation of tryptophan metabolism in the skin may lead to a reduction of AhR ligands, such as kynurenine, kynurenic acid, and FICZ [123,124,125].

In both human psoriasis samples and an IMQ-induced model of skin inflammation, FICZ-induced AhR activation ameliorates inflammatory response. Moreover, Di Meglio et al. revealed that the expression of 29 out of 41 genes upregulated in psoriasis, including inflammatory-related genes such as *IFIT, IFIT3, RSAD2* and *MX2*, was reduced after FICZ-induced AhR activation. Thus, decreased AhR activity in psoriatic skin lesions may be associated with increased expression of proinflammatory cytokines in this tissue leading to hyperinflammation [117]. Moreover, AhR activity seems to be crucial modulator of the severity of psoriasis [117]. In summary, the limited availability of endogenous AhR ligands could affect skin homeostasis regulated by this receptor.

It is not explicitly confirmed that a specific cytokine profile is responsible for the severity of skin lesions. This crosstalk between immune cells and nonhematopoietic cells involved in the inflammatory response is crucial for determining the pathogenesis of diseases such as psoriasis. However, the treatment of autoimmune inflammation is based on the modulation of the immune response [126]. An absence of AhR or blockade of its activity is associated with dysregulation of skin cell responses, mainly keratinocytes, to inflammatory stimuli. A number of inflammatory pathways are involved in the pathogenesis of psoriasis; thus, it is difficult to indicate the leading role of individual inflammatory mediators in the development of skin lesions. Recent studies indicate that the use of IL-17 blockers in an IMQ-induced psoriasis-like skin model is not sufficient to decrease the formation of skin lesions in *AHR*-deficient mice [127]. Moreover, AhR activity in the epidermal capillaries limits the recruitment of neutrophils, thus limiting the formation of skin lesions [128].

## 6. Skin Pigmentation Disorders

### 6.1. Hyperpigmentation

Hyperpigmentation of the skin is characteristic of tobacco smokers, and it may result from BaP-mediated AhR activation and enhanced melanogenesis [129]. The microphthalmia-associated transcription factor (MITF) is a major regulator of melanogenesis, which activates tyrosinase (TYR) and tyrosinase-related proteins (TYRPs). The expression of these melanogenic enzymes leads to melanin granules production [130]. The interaction of AhR with BaP or TCDD induces MITF activation, which in turn enhances TYR expression, resulting in increased melanogenesis [131]. Benzanthrone is another AhR ligand contributing to hyperpigmentation. Increased melanogenesis was observed in murine melanocytes treated with benzanthrone in vitro [132]. Skin pigmentation was also diagnosed in patients from Japan (Yusho) and Taiwan (Yucheng) after mass poisoning caused by cooking oil contaminated with PCBs and PCDFs [77,79]. Additionally, long-time exposure to high concentrations of PM2.5 may also be associated with hyperpigmentation [76].

### 6.2. Vitiligo

Vitiligo is an acquired pigmentary disorder, characterized by the loss of functioning melanocytes in skin, hair, or both. The pathogenesis of vitiligo is based on melanocyte defects, an innate immune response, and T-cell-mediated melanocyte destruction [133]. Vitiligo patients reveal a reduced expression of *AHR* in skin lesions compared to healthy controls [134]. However, furanochromones psoralen and khellin, in combination with UVA phototherapy, activate AhR, thus increasing melanogenesis [135].

AhR-mediated Treg cell differentiation and IL-10 expression may be associated with vitiligo pathogenesis, as IL-10 plays a crucial role in the development of self-tolerance [136,137]. Importantly, vitiligo is an autoimmune disease in which macrophages, T cells, cytokines, and other proinflammatory mediators play a prominent role [138]. Recent studies demonstrated increased TNF-α concentration and decreased IL-10 production in the serum of vitiligo patients [136,139]. Moreover, Tregs from *AHR*-null mice produced decreased level of IL-10 [27]. On the other hand, Taher et al. revealed that tacrolimus-induced increase in IL-10 level inhibited the degradation of melanocytes and might reduce disease symptoms [138].

The pathogenesis of this disease may be related to *AHR* − 129C > T polymorphism [139]. The T allele of this polymorphism increases the binding affinity of the SP1 transcription factor to *AHR*, thereby increasing the activity of the *AHR* promoter. Multiple TATA-less genes responsible for cell growth and immune response are controlled by SP1. *AHR* lacks TATA boxes, although its core promoter region possesses GC-rich fragments with several putative SP1 binding sites [140]. The abnormal binding affinity of the *AHR* promoter to SP1 (due to *AHR* hypermethylation or under the influence of an SP1 inhibitor) may decrease *AHR* expression. Interestingly, increased *AHR* expression was observed in carriers of the −129 T allele; thus, it could potentially be a genetic marker for vitiligo. On the other hand, −129 T allele possession is associated with higher expression of IL-10. Therefore, *AHR* − 129C > T polymorphism may be related to vitiligo by altering IL-10 production [139].

IL-22-producing cells, whose activity is dependent on AhR ligation, may also contribute to abnormal immune response underlying vitiligo [141]. Furthermore, IL-17 expression is correlated with vitiligo and may play a role in its pathogenesis [142]. However, the relationship between AhR-mediated IL-17 expression and vitiligo has not yet been stated. Similarly, the involvement of ROS in vitiligo pathogenesis remains controversial [143].

## 7. Skin Appendage Disorder: Chloracne

Chloracne is characterized by acne-like eruptions, blackheads, cysts, and pimples on the skin and may appear in response to permanent exposure to AhR ligands from polluted air, such as TCDD and PCDFs [77,78,79]. Chloracne skin lesions are located mainly on retroauricular and malar areas of the face as well as on the ear lobes and groin [80,144]. Increased *AHR* expression is observed in skin lesions of people exposed to dioxins present in polluted air [101]. Moreover, constitutive activation of AhR and excessive production of ROS may be crucial for the development of this disease [145]. Pathogenesis of chloracne is based on the accelerated process of final differentiation of keratinocytes induced by AhR agonists, although the molecular aspect of this mechanism is not fully understood [146,147].

Caputo et al. reported that exposure to high doses of TCDD contained in polluted air caused chloracne in children after the explosion in Seveso [78]. Similarly, massive poisoning of PCDFs and their derivatives induced chloracne in Japan (Yusho) [80] and Taiwan (Yucheng) [81]. Skin lesions covering over 30% of body surface area and sebaceous gland involution are observed in people exposed to very high doses of TCDD [146].

In physiological conditions, AhR activation also leads to accelerated keratinocyte differentiation [148]. However, structural stability of dioxins may be crucial for chloracne development, as endogenous AhR ligands are rapidly degraded. The key role of AhR in keratinization was confirmed in studies in on *AHR*-deficient and *AHR*-transgenic mice [73,149].

Furthermore, lipophilic dioxins can accumulate in the sebaceous glands with high *AHR* expression and might be secreted with sebum [144,150,151]. Moreover, chloracne indicates hyperkeratinization of interfollicular epidermis hair follicle cells [152]. In addition, a change in the physiology of sebocytes is observed in the form of a gradual loss of sebaceous cells and involution of sebaceous glands, which leads to cyst formation [150,153]. AhR-dioxins interaction results in hyperkeratinization of keratinocytes and transformation of sebocytes into keratinocytes [151,152,154].

Pathogenesis of chloracne is related to upregulation of the expression of particular genes and proteins. The reduced number of sebaceous glands and sebocytes may be associated with the altered metabolism of the mature sterol-binding protein (mSREBP-1), resulting from AMP-activated protein kinase (AMPK) activation [155]. On the other hand, the MAPK signaling pathway is also crucial for skin lesion formation in chloracne patients, as AhR activation in chloracne induces the activation of EGFR and MAPK [156]. EGFR and AhR compete for common coactivator p300 for their transcriptional activity. Thus, the activation of the EGFR pathway results in inhibition of AhR-mediated *CYP1A1* expression [157].

A number of compounds coordinate the course of each stage of keratinization, which includes transglutaminase-1 and -3 of ceramides and various epidermal differentiation complex (EDC) proteins [158]. TCDD indirectly accelerates keratinization by interacting with EDC molecules such as LOR and FLG [159]. The expression of LOR and FLG increases due to the interaction with TCDD and induces earlier maturation of the epidermal barrier in the skin of mouse fetuses [160]. Application of TCDD directly on hairless mouse skin resulted in hyperkeratosis, epidermal hyperplasia, and sebaceous gland metaplasia [161].

Moreover, TCDD-induced activation of AhR increases the expression of genes involved in the keratinization process. This applies especially to EDC genes and genes responsible for ceramide synthesis [162]. Inflammation in chloracne results from increased expression of cytokines (IL-6, IL-8, and IL-1a) produced by keratinocytes and sebocytes [8,84,153].

The previous studies suggested that the severity of chloracne depends on the level of dioxins in the blood [80]. AhR stimulation is associated with impaired sebocyte proliferation and impaired lipid synthesis in these cells. As a result of dioxin-induced AhR activity, sebocytes lose their characteristic phenotype; thus, inhibition of lipogenesis and a decrease in the expression of keratin 7 and the epithelial antigen membrane occur. Moreover, the transformation of sebocytes into keratinocytes is associated with increased expression of the keratinocyte-specific molecules: keratin 10 and peroxisome proliferator-activated receptor-δ (PPAR-δ) [151,152,153]. Dedifferentiation of sebocytes may depend on the activity of the AhR/Blimp1 signaling pathway. Inhibition of lipogenesis and sebaceous gland atrophy is associated with inhibition of sebocyte proliferation and reduction of c-Myc expression mediated by Blimp1 activity. Furthermore, AhR–TCDD interaction induces the AhR/Blimp1 signaling activity [152].

## 8. Skin Cancer

AhR is also associated with carcinogenesis and tissue homeostasis [163,164]. However, its role in carcinogenesis is not clearly defined, and opposite effects of AhR on tumor progression have been reported. It is hypothesized that this seemingly contradictory function of AhR in tumor progression may be partially dependent on its cell-type-specific roles in cell migration [reviewed in [165]].

There is no clear confirmation that AhR activation leads to the development of skin cancers. However, the observed procarcinogenic effects of some AhR ligands and the biological role of down-effector genes activated by this receptor suggest its involvement in carcinogenesis. Long-term observations revealed that overexposure to some synthetic AhR ligands (e.g., polycyclic aromatic hydrocarbons) or UVB may lead to premalignant lesions or skin cancer [166,167,168].

Carcinogenicity might be associated with the activity of cytochrome P-450 enzymes, as it leads to either detoxification or potential carcinogens formation. Importantly, UVB is also involved in the induction of cytochrome P-450 subfamilies, including CYP1A1 and CYP1B1, and metabolic activation and transformation of organic procarcinogens to carcinogens. Moreover, several UV-induced mechanisms may be associated with skin carcinogenesis, such as direct UVB damage to skin cell DNA, reduced apoptosis, intensified keratinocyte proliferation, and chronic skin inflammation [169]. Finally, the stimulation of AhR leads to activation of MAPK signaling which may be involved in cancer cell proliferation.

### 8.1. Squamous Cell Carcinoma

Squamous cell carcinoma (SCC) is the most frequent skin malignancy in humans [170]. Importantly, AhR was identified as one of the genetic determinants of susceptibility to SCC in humans [171]. Several procarcinogenic and proinflammatory AhR-related genes potentially involved in carcinogenesis and cancer progression are upregulated in keratinocytes exposed to UVB, including *CYP1A1*, *CYP1B1*, *COX-2*, *CXCL5*, and matrix metalloproteinases (MMPs) (reviewed in [172]). It was suggested that the AhR signaling pathway is involved in the initiation of keratinocyte-derived skin cancers induced by UVB radiation [23]. Moreover, AhR signaling may contribute to the degradation of the cyclin-dependent kinase inhibitor p27Kip1 involved in cell cycle regulation, proliferation, and apoptosis in keratinocytes [172,173,174].

### 8.2. Melanoma

Surprisingly, there have been very few studies reported on the role of AhR in melanoma promotion and progression, although *AHR* is highly expressed in melanoma cell lines [175]. Furthermore, the interactions between tumor and stroma are mediated by AhR. It was reported that although *AHR* expression in the tumor inhibits melanoma growth and metastasis, the expression of this receptor in the stroma promotes melanomagenesis. AhR might act as tumor suppressor regarding melanoma cells, as its activity was associated with decreased migration and invasion, a reduced numbers of cancer stem-like cells, and aberrant β1-integrin and caveolin 1 concentrations. Human melanoma cell lines with the highest protein level of AhR have also inhibited migration and invasion activity. Moreover, AhR protein level is reduced in human melanomas with respect to nevi lesions. It is supposed that tumor progression and metastasis depend on stromal AhR in the case of *AHR* knockdown in melanoma cells [165]. Activation of AhR signaling in the tumor microenvironment may stimulate cancer cell proliferation, and migration by enhanced expression of proangiogenic mediators and factors increased cancer cell motility, including the vascular endothelial growth factor (VEGF) and TGF-β [48,176].

On the other hand, it was reported that environmental chemicals considered as AhR agonists contribute to melanoma progression and invasion through the stimulation and activity of MMPs [177]. Another study revealed that exposure to TCDD leads to upregulation of the melanogenic pathway not only in melanocytes but also in melanoma cells. However, no stimulation of melanoma cell proliferation was observed [131].

## 9. The Role of Tryptophan-Derived AhR Ligands in Skin Homeostasis

Previous studies revealed several ligands of AhR that can be grouped as follows:

- Exogenous/synthetic ligands (i.e., TCDD, biphenyls, DMBA, methylcholanthrene, and BaP);
- Exogenous/natural compounds, found in or metabolized from dietary plants (i.e., resveratrol and other glucosinolates, flavonoids, indolcarbinols, and kynurenic acid);
- Endogenous ligands formed in the body (i.e., kynurenine, kynurenic acid, ITE, a tryptophan–cysteine dimer, and FICZ).

Several AhR agonists are derived from tryptophan, which is an essential amino acid that is considered as the strongest near-UV absorbing chromophore [65]. Thus, the role of these AhR ligands may be crucial for various processes in the skin. UV absorption by tryptophan leads to the production of several stable photoproducts that may have various biological activities. Some of these are considered as AhR ligands since conformational changes of tryptophan under exposure to UV radiation in the skin result in FICZ production [75,178]. Importantly, some other non-UV-induced tryptophan metabolites produced enzymatically in cells are also considered as AhR ligands (i.e., kynurenine and kynurenic acid) [123,179,180].

Three main ways by which tryptophan-derived AhR ligands reach the skin can be distinguished: topical application on the skin, as these ligands may be the compounds of skin care products; endogenous synthesis in cells of the skin [4]; and intragastric administration [181,182]. Furthermore, tryptophan-derived ligand activity affects various physiological and pathological processes.

### 9.1. FICZ

FICZ, a tryptophan oxidation product formed by exposure to UV or visible irradiation, binds with high affinity to AhR in mammalian cells, inducing expression of CYP1A1 [183]. UVB is the most efficient in FICZ formation from aqueous tryptophan, whereas visible light and UVA induce FICZ production with lower yields [184]. FICZ has a very high affinity for AhR but is quickly and efficiently degraded in cells by AhR-induced CYP1A1, CYP1A2, and CYP1B1, giving it low intracellular levels [185,186]. Importantly, FICZ has been found to be physiologically relevant in human skin [187]. However, the biological role of this tryptophan metabolite in physiological and pathological processes in the skin has not been fully studied. It was revealed that direct FICZ-mediated AhR activation alleviates inflammation in both human psoriasis samples and a mouse model of psoriasis-like skin lesions [117]. The FICZ–AhR interaction activates the AhR/ROS signaling pathway and increases the expression of inflammatory mediators (IL-1A, IL-1B, and IL-6) and, thus, may be associated with the dangerous effects of exposure to UVB radiation [8].

FICZ reveals a photosensitizing effect on keratinocytes. The simultaneous exposure to FICZ and UVA radiation induces apoptosis of keratinocytes due to caspase 3 activation and heat shock protein 70 (HSP70) production [183,184]. Moreover, FICZ reduces TGF-β-mediated collagen formation in human dermal fibroblasts [187,188]. These data indicate that FICZ may be responsible for the effect of photoaging after UVB exposure.

On the other hand, FICZ limits the production of IL-17 and IL-22 in skin lesions and reduces inflammation in dermatitis model [98,117]. Moreover, FICZ-mediated AhR activation is associated with increased expression of EDC, such as FLG and LOR [71]. FICZ promotes wound healing by increasing keratinocyte migration due to the activation of the MEK/ERK pathway in an AhR-independent manner [105]. Cell migration is supported by FICZ even in the conditions of AHR knockdown by small interfering RNAs (siRNAs) or an AhR inhibitor [105]. Therefore, inflammatory cell migration may result directly from interactions between FICZ and the TGF-β/ERK signaling pathway. However, the effect of FICZ may be associated with other molecular mechanisms stimulated by injury. These results shed a new light on the role of FICZ in skin homeostasis. Nevertheless, the mechanism of FICZ-mediated keratinocyte migration may be relevant to managing the treatment of skin wounds.

Mengoni et al. reported that *AHR* expression strictly correlates with the degree of dedifferentiation in both human melanoma samples and human and mouse melanoma cell lines [189]. Moreover, in the inflammatory environment, FICZ-mediated AhR activation induces the phenotypic switch of melanoma cells into the dedifferentiated state [189]. In addition, AhR-induced suppression of E-cadherin expression and induction of MMP activity resulted in reduced cell adhesion and increased cell motility [177,190]. Taken together, these data indicate that AhR activity may promote invasive features of tumor cells.

### 9.2. Kynurenine

Kynurenine, a key metabolite of the main route of tryptophan catabolism, is an endogenous agonist of AhR [191]. Although kynurenine activates the AhR using classical response genes such as *CYP1A* [191], it was previously revealed that kynurenine plays a more important role in AhR-dependent immunological responses rather than in the metabolism of xenobiotics. In a dose-dependent manner, kynurenine upregulates the expression of immunosuppressive genes, such as *TGFB1* and *IDO1* [192,193]. Kynurenine regulates T-cell differentiation and induces immunosuppressive strategies in cancer cells [124,191]. Moreover, kynurenine may display an immunosuppressive activity; thus, it takes part in disease tolerance pathways and represents a link between tryptophan catabolism and the AhR signaling pathway [192].

Although the impact of kynurenine on cancer cell proliferation is not fully understood, recent studies indicate that kynurenine activity is related to anticancer immune response. Kynurenine is produced by the tryptophan catabolizing enzymes, indoleamine 2,3-dioxygenase (IDO) and tryptophan 2,3-dioxygenase (TDO), in several types of cancer, including melanoma, to promote immune evasion [124,194]. Moreover, TCDD, one of the synthetic AhR ligand, determines tumor immunity as it promotes IDO activation, leading to kynurenine formation. *IDO* is constitutively expressed by many tumors and promotes immunosuppressive mechanisms due to depletion of tryptophan. Moreover, IDO promotes the formation of several tryptophan metabolites such as kynurenine with immunosuppressive activity. It was reported that *IDO* expression is associated with unfavorable prognosis in patients with various malignancies (reviewed in [195]). Importantly, expression and activity of IDO 1 and 2 are controlled by inflammatory mediators [196].

Similarly to tryptophan derivatives, AhR activity is associated with immune response regulation, as it was previously demonstrated in fibroblasts, endothelial cells, and macrophages [197,198,199]. Bessede et al. reported that tryptophan metabolites—AhR interaction contributes to the activation of Scr kinase, thus promoting IDO1 phosphorylation [192]. Furthermore, TGF-β expression is blocked, as kynurenic acid cannot induce its activation without IDO1. TGF-β is a major immune tolerance indicator; thus, AhR-mediated IDO1 phosphorylation affects immune response [192].

### 9.3. Kynurenic Acid

Kynurenic acid, a product of tryptophan metabolism enzymatically formed from kynurenine, is a natural ligand for AhR. Kynurenic acid is produced by kynurenine aminotransferases (KATs), which promotes L-kynurenine transamination. Moreover, the presence of ROS allows the direct transformation of tryptophan or kynurenine into kynurenic acid (reviewed in [179,196]). Kynurenic acid in nanomolar concentrations is an efficient agonist for the human AhR inducing IL-6 production and xenobiotic metabolism in cells [123]. Nevertheless, the role of kynurenine pathway metabolites in AhR-mediated skin homeostasis remains unclear. Recent studies indicate that AhR-kynurenic acid interaction may be relevant for maintaining the immunosuppressive microenvironment in several cancer types [179,200].

It has been revealed that kynurenic acid has various biological activities, including neuroprotective, anticonvulsant, anti-inflammatory, antioxidant, and antiulcer activity (reviewed in [179,196]). Importantly, kynurenic acid also has antiproliferative and antimigratory properties against various types of cancer cells (reviewed in [179]) by inhibition of signaling pathways (MAPK, PI3K/Akt) and overexpression of cell cycle regulatory proteins (p21Waf1/Cip1) [119,201]. Moreover, a recent study confirmed the biological activity of kynurenic acid towards melanoma A375 and RPMI-7951 cells [202].

Kynurenic acid is formed endogenously and is present in almost all human body fluids and tissues (reviewed in [179]) Importantly, kynurenic acid is also present in several products of human diet [181,182]. The intragastrically administered KYNA is absorbed and transported to peripheral organs via the bloodstream [203]. The role of kynurenic acid in the skin is not fully studied. It was reported that kynurenic acid is phototoxic for erythrocytes and glia cells, but no specific studies regarding skin cells have been performed [204,205].

**Figure 3:**
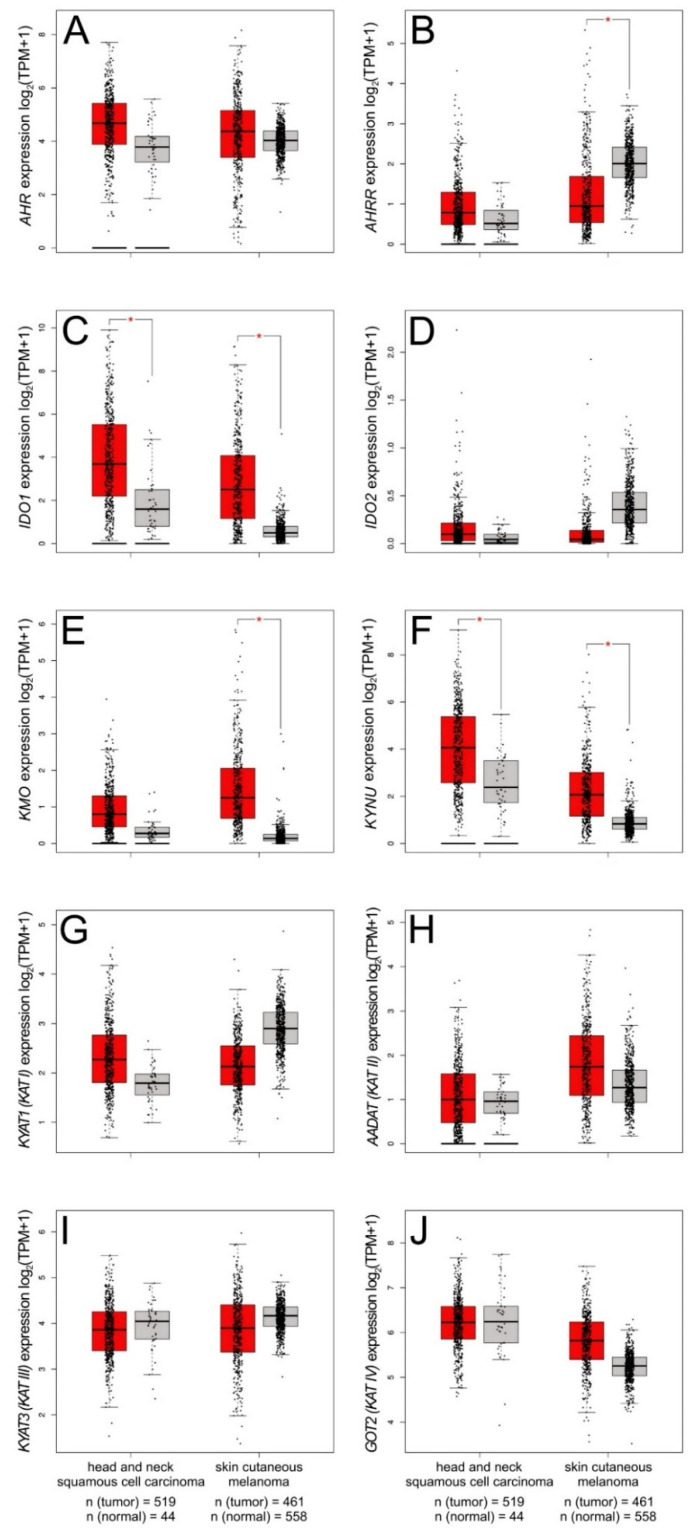
Expression pattern of genes encoding *AHR* (**A**), *AHRR* (**B**), tryptophan catabolizing enzymes (*IDO1* (**C**), *IDO2* (**D**), *KMO* (**E**), *KYNU*(**F**)) and kynurenine aminotransferases (*KAT I–IV* (**G**–**J**)) in human head and neck squamous cell carcinoma and skin cutaneous melanoma. Expression of *KYAT1* (*KAT I*), *AADAT* (*KAT II*), *KYAT3* (*KAT III*), *GOT2* (*KAT IV*) was analyzed. Significant downregulation of *AHRR* expression was observed in skin cutaneous melanoma (**B**). Both head and neck squamous cell carcinoma and skin cutaneous melanoma showed significantly higher expression of *IDO1* (**C**). Moreover, significantly upregulated expression of *KMO* was found in skin cutaneous melanoma (SKCM) (**E**). Significantly upregulated *KYNU* expression was observed in both HNSC and SKCM (**F**). GEPIA2 was queried for skin cancers: human head and neck squamous cell carcinoma and skin cutaneous melanoma [207]. Differences in gene expression levels were studied using ANOVA. * *p* < 0.01 and fold-change threshold (|Log2FC| cutoff) of 1.

Although *AHR* expression levels do not differ significantly in various types of skin cancer (Figure 3A), we observed a significant downregulation of *AHRR* expression in skin cutaneous melanoma (SKCN) (Figure 3B). Vogel et al. report that the upregulated AhRR expression inhibits the AhR-mediated antiapoptotic response in mouse embryonic fibroblasts [206]. As AhRR tends to play a significant role in suppressing inflammation, the downregulated *AHRR* expression may promote tumor growth.

Interestingly, the expression of genes encoding tryptophan catabolizing enzymes (e.g., *IDO1* and *KYNU*) is significantly upregulated in two types of skin cancer: head and neck squamous cell carcinoma (HNSC) and SKCN (Figure 3).

Although the reason for this phenomenon has not yet been revealed, a few hypotheses seems to be reliable and feasible. The kynurenine pathway is a major metabolic pathway involved in the formation of key a coenzyme, nicotinamide adenine dinucleotide (NAD+). As cancer cells display increased energy requirements, overexpression of *IDO1* and *KYNU* may arise from the need of an additional source of energy NAD+. On the other hand, it cannot be ignored that the increased activation of KYNU may be caused by the need to reduce the amount of kynurenine or kynurenic acid, which may have a negative effect on cancer cells.

Theate et al. indicate that the expression of *IDO1* may act like a negative prognostic marker in various cancer types, including melanoma and carcinomas of the cervix, bladder, kidney, and lung [208]. Moreover, AhR regulates the expression of *IDO1* and *TDO*. Regarding the tumor microenvironment, a decreased level of tryptophan caused by IDO1 and TDO activity may result in loss of immune function through the suppression of antigen-specific T-cell response and stimulation of DC-mediated immune tolerance [124]. Thus, declined effectiveness of the anticancer immune response, resulting from deregulation of the kynurenine pathway, may be associated with cancer progression. Moreover, as the activation of the IDO/kynurenine/AhR pathway is associated with the resistance to immune checkpoint blockade, AhR may be involved in therapy resistance [209].

### 9.4. Skin Microbiome Metabolites

The epidermis may be colonized by various species of commensal microbes. For instance, lipophilic yeasts *Malassezia* are capable of converting tryptophan into indole compounds, some of which are AhR agonists. *Malassezia* furfur and *Malassezia globosa* colonize the skin of approximately 80% of the healthy population. However, their impact on skin physiology is controversial [12,210].

The activity of tryptophan-derived AhR agonists produced by *Malassezia* is associated with the hyperproliferation in seborrheic dermatitis and altered inflammatory in pityriasis versicolor [210]. Moreover, *Malassezia* metabolites affect cell cycle regulation and DNA repair, thus increasing the risk of skin cancer. Gaitanis et al. also reported that AhR ligands produced by Malassezia change ROS production and suppress the inflammatory response [211].

## 10. Conclusions

Previous studies confirmed at least a partial role of AhR in the pathogenesis of various skin diseases, including inflammatory diseases, skin pigmentation disorders, and cancer [84,89,154]. However, the function of AhR is complex as the outcome of AhR activation depends on the type of cell and ligand [13,15]. Furthermore, many different biological responses to AhR stimulation or inhibition in the skin are observed [56]. Most of the reported data are focused on the immunological and oncological effect of AhR stimulation. However, AhR ligation may induce excessive expression of proinflammatory cytokines and ROS production, leading to inflammatory disease development or carcinogenesis [55]. On the other hand, AhR-agonist-mediated activity may affect the differentiation of Treg cells, thus promoting immune tolerance [126,127]. Therefore, to determine the physiological mechanism of AhR and its role in skin disease development, more data are needed from both basic and clinical studies.

Importantly, tryptophan derivatives are a large group of AhR ligands that may potentially play a role in the pathogenesis or treatment of many skin diseases [7,169]. They are produced by enzymatic reactions or due to UV radiation in various skin cells; thus, skin is constantly exposed to tryptophan-derived AhR ligands. Additionally, some of them are present in herbs and plant extracts commonly used in skincare and treatment. However, their biological role requires further examination. In future studies, the involvement of tryptophan-derived AhR ligands in the initiation and progression of skin diseases should be clarified. The question of whether tryptophan-derived AhR ligands should be used in the prevention of skin diseases or whether we should avoid contact with them due to their negative impact on disease progression remains without a clear answer.

## Figures and Tables

**Table 1:** The effect of AhR on selected cellular processes.

Alterations in Cellular Functions		Biological Effect	Type of Cell/ Mouse Model	Reference
Cell metabolism		AhR stimulates the expression of enzymes involved in drug metabolism (e.g., CYP1A1, CYP1A2, and CYP1B1).	*AHR*-deficient mice	[39]
Cell proliferation	Inhibition	AhR-dependent mechanisms induce the expression of genes for the CDK inhibitory factors p27 ^Kip1^ and p21 ^Waf1/Cip1^ leading to an inhibition of CDKs and Rb inactivation.	5L cells	[40]
			HUVEC	[41]
		AhR binds directly to hypophosphorylated Rb and prevents its phosphorylation by cyclin-dependent kinases (CDKs).	LNCaP cells	[42]
			BP8 5L HEK293	[43]
		AhR interacts directly with E2F, promoting the expression of S-phase-specific genes.	Hepa-1c1c7 MCF-7	[44]
		*AHR* silencing inhibits cell cycle progression and proliferation.	HepG2	[45]
	Stimulation	*AHR* silencing stimulates cell cycle and proliferation of cells.	MCF-7	[45]
		AhR acts as potent transcriptional coactivator of E2F1-dependent transcription.	A549	[46]
		AhR induce the expression of SOS1, accelerating cell proliferation.	HepG2	[47]
Cell migration *		AhR promotes the increased formation of cytoskeleton stress fibers and reduction of lamellipodia formation, and decreases migration of fibroblasts in *AHR* knockdown mice.	T-FGM-*AHR*−/− myofibroblasts	[48]
		TCDD-induced AhR activity promotes cell motility and cytoskeleton remodeling.	MCF-7 HepG2	[49]
		Flavin, an AhR agonist, induces the inhibition of breast cancer cell growth and motility.	MDA-MB-231 T47D	[50]
		Omeprazole, an AhR agonist, decreases breast cancer cell invasion and suppress metastasis.	MDA-MB-231	[51]
		*AHR* knockout reduces cell migration due to heregulin signaling activation in breast cancer cells displaying HER2 overexpression.	MCF-7	[52]
		Hyperactivation of AhR accelerates cell migration of oral squamous cell carcinoma cells, while AhR inhibition reduces migration of these cells.	HSC-3 CAL27	[53]
**Regulation of Signaling Pathways and Nuclear Receptors**				
NF-κB signaling pathway		TCDD-mediated AhR activation stimulates the transcription of inflammatory genes within the NF-κB signaling pathway, e.g., *IL 8.*	U937 macrophages	[54]
		AhR-mediated *IL 17A* and *CCL20* transcriptional activation is dependent on RelB activity.	B6 mice	[25]
Nuclear factor-erythroid 2-related factor-2 (Nrf2) signaling pathway		AhR promotes the expression of antioxidant enzymes such as glutathione S-transferases and NAD(P)H quinone dehydrogenase 1 (NQO1).	NHEK	[55,56]
Calcium-dependent signaling pathways		AhR ligands TCDD and polycyclic aromatic hydrocarbons (PAH) are able to produce rapid and sustained calcium influx.	Hepa-1	[57]
		AhR is involved in stimulating the 35-cyclic adenosine monophosphate (cAMP), protein kinase C (PKC), and protein kinase A (PKA) activity, promoting the inflammatory response to TCDD.	3T3-L1	[58,59]
Hypoxia-induced factor (HIF)		Under hypoxic conditions, HIF1α binds to ARNT, limiting its bioavailability for AhR and inhibiting AhR transcriptional response.	HepG2 HaCaT	[60]
		Low O_2_ conditions stabilize HIF1α and HIF2A, the absence of which impairs the expression of the HIF-targeted gene encoding filaggrin; thus, keratinocyte terminal differentiation and epidermal barrier formation are impaired.	*HEK* *Krt14-Cre*+ mice	[61]
		TCDD-mediated AhR activation improperly expresses R-Spondin1, which mediates through LRP6 to activate the Wnt/β-catenin signaling;Activation of Wnt/β-catenin results in the stabilization of β-catenin, which in turn causes the misexpression of various Wnt target genes, resulting in the inhibition of tissue regeneration.	Zebrafish caudal fin regeneration model	[62]
Estrogen and retinoid receptors		AhR ligand TCDD stimulates the expression of a gene product that inhibits estrogen receptor α (ERα)-dependent induction of transcription.	BG1	[63]
		CYP1A/1B induction increases estrogen catabolism.	MCF-7	Reviewed in [28,64]

* Although AhR activity may influence cell migration and invasion, the ability of the AhR to drive tumor growth is mostly tissue specific. AhR—aryl hydrocarbon receptor; CDK—cyclin-dependent kinase; Rb—retinoblastoma protein; E2F—a group of transcription factors, which are downstream effectors of Rb; SOS1—son of sevenless 1; TCDD—2,3,7,8-tetrachlorodibenzo-p-dioxin; IL17A—interleukin 17A; CCL20—chemokine (C-C motif) ligand 20; NQO1—NAD(P)H quinone dehydrogenase 1; PAH—polycyclic aromatic hydrocarbons; cAMP—3′5′-cyclic adenosine monophosphate; PKC—protein kinase C; PKA—protein kinase A; ARNT—aryl hydrocarbon receptor nuclear translocator; LPR6—LDL receptor-related protein 6; ERα—estrogen receptor α; NF-κB—nuclear factor kappa-light-chain-enhancer of activated B cells; HIF—hypoxia-induced factor; Nrf2—nuclear factor-erythroid 2-related factor-2.

**Table 2:** The AhR signaling pathway mediates antioxidative signals in response to different substances, e.g., herbal medicines and flavonoids.

Substance	Outcome	Cell Type	References
Ketoconazole	Activation of antioxidative Nrf2 and NQO1 pathways; Anti-inflammatory effect mediated by TNF-α inhibition; Inhibition of BaP-mediated ROS and IL-8 production (cytoprotective effect); AhR activation without ROS production.	NHEK	[55]
*Bidens pilosa*	Inhibition of TNF-α and BaP-mediated ROS production; Activation of antioxidative Nrf2 and NQO1 pathways.	Human dermal endothelial cells	[86]
Epigallocatechin gallate	Activation of antioxidative Nrf2 and NQO1 pathways; Downregulation of AhR and CYP1A1 expression.	Primary vascular endothelial cells	[87]
Quercitrin	Inhibition of UVB-mediated ROS production; Reduction of UVB-mediated oxidative DNA damage.	JB6 cells	[88]
Quercetin, kaempferol	Reduction of BaP-mediated increased expression of Nrf2; Counteraction of BaP-mediated suppression of AhRR.	Caco2	[89]
Cinnamaldehyde	Inhibition of AhR activation; Activation of antioxidative Nrf2 and NQO1 pathways.	HaCaT	[11]
Cynaropicrin (*Cynara scolymus*)	Activation of antioxidative Nrf2 and NQO1 pathways; Inhibition of ROS production in cells after exposure on UVB radiation; Inhibition of proinflammatory cytokine (IL-6 and TNF-α) production in cells after exposure on UVB radiation.	NHEK	[10]
*Opuntia ficus indica*	Activation of antioxidative Nrf2 and NQO1 pathways; Induction of *FLG* and *LOR* expression.	HNEK	[9]
Hesperetin	Inhibition of AhR transactivation; Inhibition of AhR downstream gene expression (*CYP1A1*, *CYP1A2*, and *CYP1B1*).	MCF-7	[90]
Quercetin, resveratrol, curcumin	Induction of CYP1A1 in an indirect manner by inhibiting the metabolic turnover of FICZ.	HaCaT	[91]

AhR—aryl hydrocarbon receptor; Nrf2—nuclear factor-erythroid 2-related factor-2; NQO1—NAD(P)H quinone dehydrogenase 1; TNF-α—tumor necrosis factor alpha; BaP—benzo[a]pyrene; ROS—reactive oxygen species; AhRR—aryl hydrocarbon receptor repressor; FLG—filaggrin; FICZ—6-formylindolo[3,2-b]carbazole.

## Abbreviations

AD	atopic dermatitis
AhR	aryl hydrocarbon receptor
AhRR	aryl hydrocarbon receptor repressor
Akt	protein kinase B
AMPK	AMP-activated protein kinase
ARNT	aryl hydrocarbon receptor nuclear translocator
ATP	adenosine triphosphate
BaP	benzo[a]pyrene
cAMP	3′5′-cyclic adenosine monophosphate
CCL17	chemokine (C-C motif) ligand 17
CCL22	chemokine (C-C motif) ligand 22
CDK	cyclin-dependent kinase
DC	dendritic cell
DMBA	7,12-dimethylbenz[a]anthracene
EDC	epidermal differentiation complex
EGFR	epidermal growth factor receptor
ER	estrogen receptor
ERK	extracellular signal-regulated kinase
FICZ	6-formylindolo[3,2-b]carbazole
FLG	Filaggrin
HIF	hypoxia-induced factor
HNSC	head and neck squamous cell carcinoma
HMOX1	heme oxygenase 1
HSP	heat shock protein
IaId	indole-3-aldehyde
IDO	indoleamine 2,3-dioxygenase
IFN-γ	interferon gamma
ITE	2-(1H-Indol-3-ylcarbonyl)-4-thiazolecarboxylic acid methyl ester
KAT	kynurenine aminotransferases
KLF6	Kruppel-like factor 6
KYNA	kynurenic acid
KYNU	kynureninase
LC	Langerhans cell
LOR	Loricrin
LPR6	LDL receptor related protein 6
MAPK	mitogen-activated protein kinase
MITF	microphtalmia-associated transcription factor
Msrebp-1	mature sterol-binding protein
NAD	nicotinamide adenine dinucleotide
NF-κB	nuclear factor kappa-light-chain-enhancer of activated B cells
NQO1	NAD(P)H quinone dehydrogenase 1
Nrf2	nuclear factor-erythroid 2-related factor-2
PAH	polycyclic aromatic hydrocarbons
PBMC	peripheral blood mononuclear cell
PCB	polychlorinated biphenyls
PCDD	polychlorinated dibenzo-p-dioxins
PCDF	polychlorinated dibenzofurans
PI3K	phosphoinositide 3-kinase
PKA	protein kinase A
PKC	protein kinase C
PPAR-δ	peroxisome proliferator-activated receptor-δ
PTD	photodynamic therapy
Rb	retinoblastoma protein
ROS	reactive oxygen species
SCC	squamous cell carcinoma
siRNA	small interfering RNA
SKCN	skin cutaneous melanoma
SOS1	son of sevenless 1
STAT	signal transducer and activator of transcription
TCDD	2,3,7,8-tetrachlorodibenzo-p-dioxin
TDO	tryptophan 2,3-dioxygenase
TGF-β	transforming growth factor beta
TNF-α	tumor necrosis factor alpha
Treg	T regulatory cell
TRP	tryptophan
TSLP	thymic stromal lymphopoietin
TYR	tyrosinase
TYRP	tyrosinase-related protein
VEGF	vascular endothelial growth factor
XAP2	the hepatitis B virus X-associated protein 2
XRE	xenobiotic-responsive element
